# Heterogeneous Breast Phantom Development for Microwave Imaging Using Regression Models

**DOI:** 10.1155/2012/803607

**Published:** 2012-04-08

**Authors:** Camerin Hahn, Sima Noghanian

**Affiliations:** Department of Electrical Engineering, University of North Dakota, 243 Centennial Drive, Uspon Hall II, Room 160, Grand Forks, ND 58202-7165, USA

## Abstract

As new algorithms for microwave imaging emerge, it is important to have standard accurate benchmarking tests. Currently, most researchers use homogeneous phantoms for testing new algorithms. These simple structures lack the heterogeneity of the dielectric properties of human tissue and are inadequate for testing these algorithms for medical imaging. To adequately test breast microwave imaging algorithms, the phantom has to resemble different breast tissues physically and in terms of dielectric properties. We propose a systematic approach in designing phantoms that not only have dielectric properties close to breast tissues but also can be easily shaped to realistic physical models. The approach is based on regression model to match phantom's dielectric properties with the breast tissue dielectric properties found in Lazebnik et al. (2007). However, the methodology proposed here can be used to create phantoms for any tissue type as long as *ex vivo*, *in vitro*, or *in vivo* tissue dielectric properties are measured and available. Therefore, using this method, accurate benchmarking phantoms for testing emerging microwave imaging algorithms can be developed.

## 1. Introduction

A major problem in developing a microwave imaging systems for tumor detection is the lack of standards in benchmarking these systems using a dielectrically accurate human analog. Many researchers use objects that are physically or dielectrically dissimilar to human tissue [1–3]. There are a few phantoms based on heterogeneous and dispersive breast tissue dielectric properties presented in the literature [4–7]. It is important to be able to match the dielectric properties for a variety of tissues over a span of frequency band. Therefore, a systematic method for creating a mixture with desired dielectric properties was needed. This method should provide a procedure to find the required amount of each material in the mixture to match the desired permittivity and conductivity for a given frequency band. We present an approach based on regression model to create mixtures that are both dielectrically similar and represents accurate physical and physiological properties of breast tissues.

Multivariable regression analysis was used to approximate the dielectric properties based upon the contents of a chemical mixture, due to a nonlinear relationship between chemical content of mixtures and their dielectric properties. The regression model for this process was generated by analyzing several mixtures and the changes in their dielectric properties. To produce these data sets, four material types with varying masses were considered and their conductivity and permittivity were measured. Multivariable regression analysis was performed on this set of mixtures, and the results were used to predict a mixture with desirable dielectric properties. The measured results and chemical contents were added to the database and used to generate the regression equations. This process was repeated until a suitable mixture with broadband dielectric match was developed.

## 2. Material Selection and Methodology

Our preliminary research [5] revealed that propylene glycol, distilled water, and oil could be used to create a phantom that mimics breast tissue permittivity well; however, the conductivity was not close enough. We used distilled water and propylene glycol as a starting point, but we needed to make changes to the phantom to match the dielectric properties to different breast tissues.

**Figure 1:**
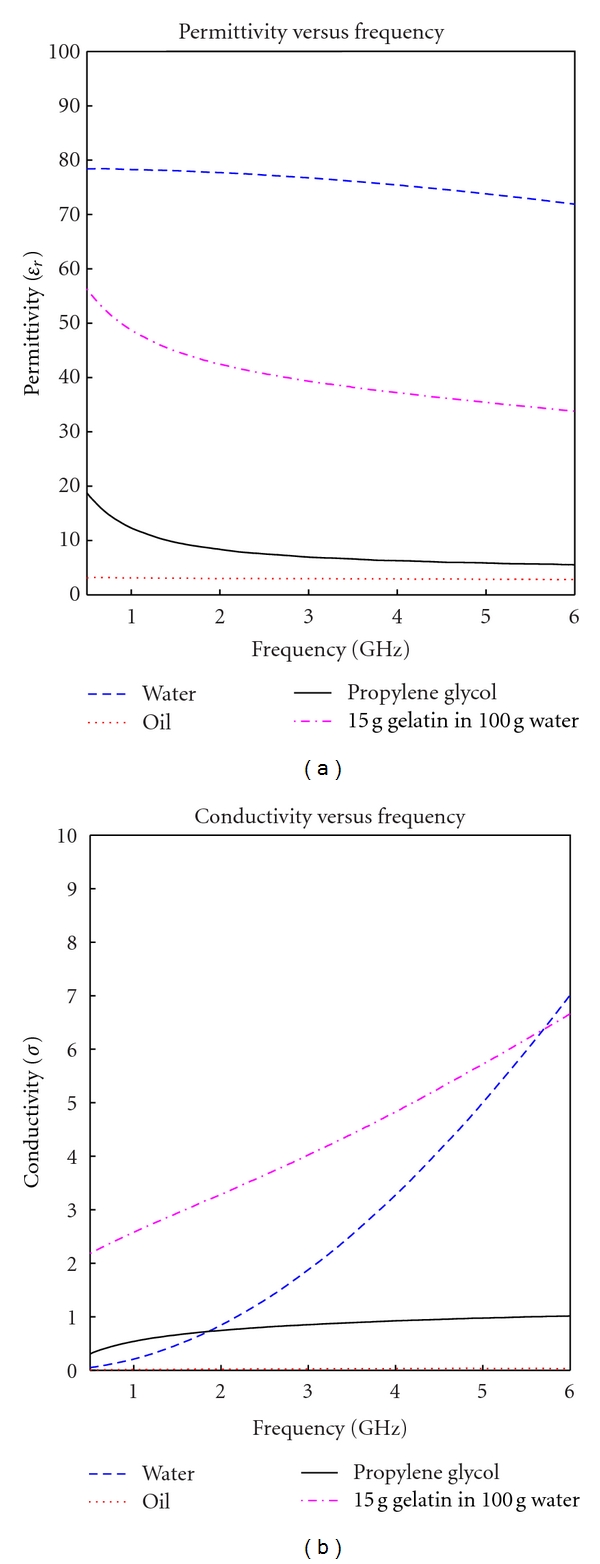
Independent measurements of different material types.

In previous work it was found that the dielectric properties can be controlled by changing the amount of water [8], methanol, and ethanol [9]. We used these ideas and applied them to more complex mixtures. By varying the water contents one is able to control either conductivity or permittivity; however, both parameters are affected by adding materials, for these reasons more than two types of materials must be used to control the dielectric properties within a frequency spectrum. These facts lead us to the belief that varying the materials from previous research [5] may result in a better match for both conductivity and permittivity. Before combining mixtures the dielectric properties of each of the materials were measured using Agilent 8570 high performance dielectric probe and Agilent E5071C network analyzer, the results of these measurements are shown in Figure 1. In this paper, all permittivity graphs represent relative permittivity (*ε*_r_).

From Figure 1 one can see that these materials have very different dielectric properties. It is expected that the high permittivity of water will allow for a close match for creating a phantom with high permittivity, while the low conductivity of propylene glycol and oil allow for reduction of conductivity. This supports the original speculation that a mixture with low conductivity can be created from these materials.

## 3. Regression Analysis

Multivariable regression is a form of regression analysis that tests the response of one or more dependent properties based upon changes in several other independent properties [10, 11]. For this study the independent variables were considered to be the mass of each material used in the mixture: including water, oil, propylene-glycol, and gelatin. The dependent variables were considered to be conductivity and permittivity at discrete frequencies across the spectrum of interest. These dependent variables are chosen due to the dispersive nature of human tissue, that is, the dielectric properties dependence on frequency [12, 13]. To begin regression the analysis-based data set is required. For this data set a group of sixteen mixtures were created by varying the mass of each material in the mixture. For each material used, bounds were set to provide the desired material properties. The regression equations are only valid within this range where data has been taken. The mixtures used to obtain the initial data set were derived from these extremes and are listed in Table 1. Each mixture in this set was measured across a frequency spectrum and the resulting data was compiled for multivariable regression analysis.

Regression analysis is a complex and repetitive task. Many software packages are available for these processes [14]. The analysis of our data was completed in Minitab. After processing the data, Minitab organized the data in a manner that was simple to understand. 

**Figure 2:**
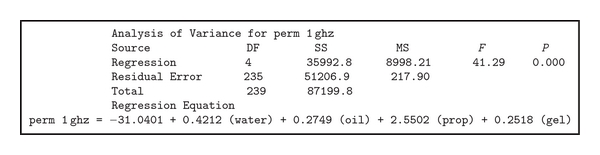
Textual output from Minitab.

When completing a regression analysis of a data set in Minitab, an output similar to one shown in Figure 2 is generated. This output provides very important information regarding the regression data generated by Minitab, including: the dependent variable (e.g., permittivity at 1 GHz: perm 1 ghz), degrees of freedom (DF), mean squared (MS), sum of squares (SS), power of regression equation (*P*), location of the power in a normalized *F* distribution (*F*), residual error, and regression information [10, 11]. This information provides some insight into how well the regression equation fits the data set. DF is the number of data points used to calculate the information minus one. SS is the sum of all of the data points squared; this is most useful in the residual error calculation due to the fact that the error can be positive or negative. MS is the mean squared of the residual error or regression data points [11] (high error due to a simple linear equation shows this equation is not adequate to model this data set in this case). *F* and *P* values are directly related; *F* value is a part of a normalized probability distribution used to find the *P* value of either a variable in the equation or the equation itself. The *P* value describes the strength of the relationship between the data sets and the regression equation as well as terms in the equation [11]; whereas, the regression equation contains the statistical relationship between the dependent and independent variables. The *P* value gives the strength of a hypothesis, the lower the *P* value the stronger the hypothesis. Typically *P* values of less than 0.005 are acceptable. This is useful for eliminating variables from the equation.

**Figure 3:**
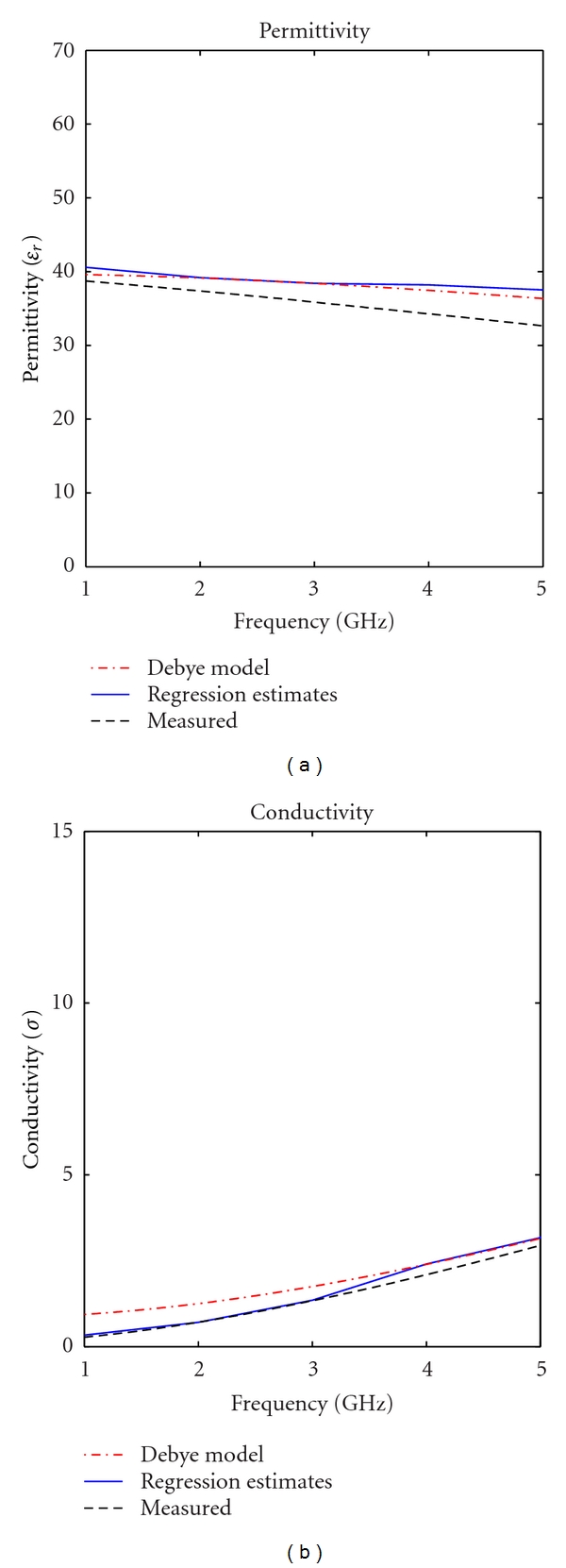
Depiction of regression model prediction for glandular phantom. The Debye model information was obtained from [12].

In our tests, the *P* values were zero for all cases (within the accuracy of the software), thus the relationship between the generated formulas and the actual data is very strong. However, after comparing these equations with previously measured mixtures and analyzing residual errors (as listed in Table 1), it was found that simple linear model, such as the one shown in Figure 2, could not accurately model the changes in this mixture. As recommended by Navidi [10], the possibility of codependence of these materials was investigated. The results of this investigation conclusively showed that co-dependent regression variables were necessary for predicting a mixture's dielectric properties. The results of our regression equation for predicting the dielectric properties are shown in Figure 3. As one can notice, the equations did not perfectly predict the actual properties of the mixture. As more data was gathered, the equations became more accurate. The form of the co-dependent equation is shown in (1):

(1) $$\begin{array}{cc} \text{property} & =a_{\text{constant}}+a_{1}m_{\text{water}}+a_{2}m_{\text{oil}}+a_{3}m_{\text{prop}}+a_{4}m_{\text{gel}}\\ & \;+b_{1}m_{\text{water}}m_{\text{oil}}+b_{2}m_{\text{water}}m_{\text{prop}}+b_{3}m_{\text{water}}m_{\text{gel}}\\ & \;+b_{4}m_{\text{oil}}m_{\text{gel}}+b_{5}m_{\text{oil}}m_{\text{prop}}+b_{6}m_{\text{prop}}m_{\text{gel}}\\ & \;+c_{1}m_{\text{water}}m_{\text{oil}}m_{\text{prop}}+c_{2}m_{\text{water}}m_{\text{oil}}m_{\text{gel}}\\ & \;+c_{3}m_{\text{water}}m_{\text{prop}}m_{\text{gel}}+c_{3}m_{\text{oil}}m_{\text{prop}}m_{\text{gel}}\\ & \;+d_{1}m_{\text{water}}m_{\text{oil}}m_{\text{prop}}m_{\text{gel}}+\text{constant}, \end{array}$$

where

(2) $$\begin{array}{c} 40\;g<m_{\text{water}}<80\;g,\\ 10\;g<m_{\text{oil}}<50\;g,\\ 2\;g<m_{\text{prop}}<7\;g,\\ 5\;g<m_{\text{gel}}<10\;g, \end{array}$$

where *a*_*i*_, *b*_*i*_, *c*_*i*_, and *d*_*i*_ are the regression coefficients given by regression analysis. *m*_water_, *m*_oil_, *m*_prop_, and *m*_gel_ are the masses for water, oil, propylene glycol, and gelatin, respectively. Equation (1) also provides the bounds for each material. These bounds are set based on to the range for which data has been taken. This range was determined through experiments performed previous to data collection. When the materials are outside of the given ranges the physical properties of the mixture begins to deviate from the desirable values, that is, the mixture may become either fluid or physically too viscous to mix.

For this study, MATLAB [15] was used to process the regression equations and make predictions for various mixtures. Predictions were made by varying the amount of each material in the mixture, within the limits given in (1), and calculating the dielectric properties using the regression equation. Invalid cases requiring negative mass or extreme amounts of each material were omitted from the solution provided by MATLAB. Many mixtures were considered and processed. For each mixture the amount of each of four main substances varied within the acceptable region as shown in (1). The predicted permittivity and conductivity of each mixture at different frequencies were subtracted from the desired ones given by the Debye model [12] and the results were recorded in an error matrix. The error was minimized by changing the amount of each four main substances. The mixture that provides minimum error was chosen.

## 4. Results of Multiple Iterations

**Figure 4:**
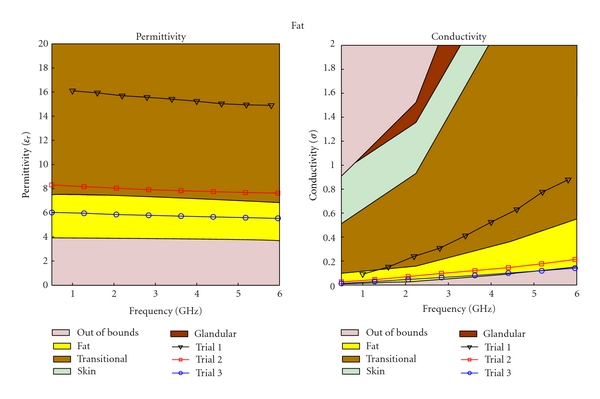
Regression results for fat phantom.

**Figure 5:**
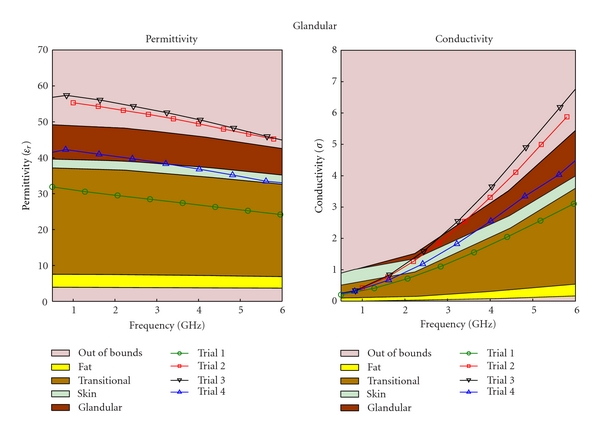
Regression results for fibroglandular phantom.

**Figure 6:**
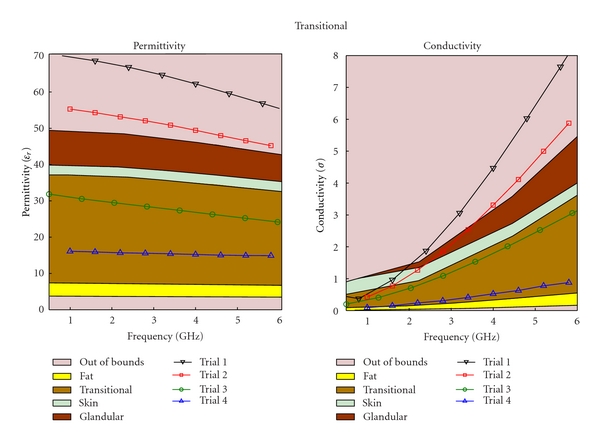
Regression results for transitional phantom.

**Figure 7:**
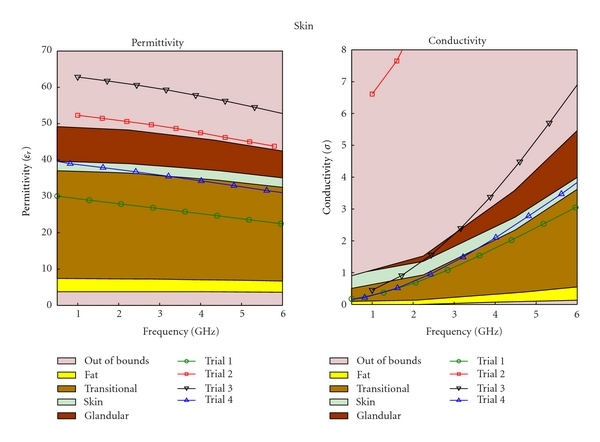
Regression results for skin phantom.

**Figure 8:**
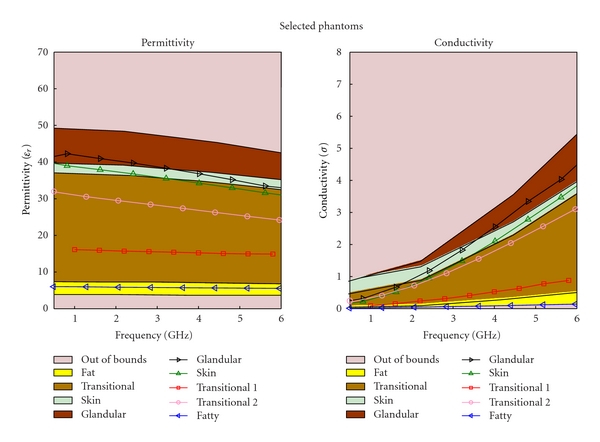
Final results for the selected phantom.

The regression process is an iterative process; as more mixtures are created, and more data sets are collected, better regression models are obtained. After three iterations, the regression equation became substantially different from the initial equation. Different types of tissue phantoms (fat, transitional, fibroglandular, and skin tissues) required different number of trials; however, each trial for the four tissue types had the same data sets, The results for each of these trials are plotted in Figures 4, 5, 6, and 7, for fat, fibroglandular, transitional, and skin tissues, respectively. Acceptable ranges for each tissue type are shown with different background colors and shades. These correspond to the measured tissue properties in [12, 13]. One can see how in general the error decreases for each trial. As the regression process progressed the mixtures properties became closer to the desired properties.  Figure 8 shows the measurement results of the selected tissue mimicking phantoms. In most cases the latest trial is the most accurate one. For the selected phantoms, the mass of different materials including formalin and surfactant for each tissue phantom are given in Table 2. Since formalin and surfactant were in small amounts, their effects on the dielectric properties were ignored and were not used as variables in the regression model. For each phantom 9 measurements were performed. The standard deviation (STD) at each frequency and for each phantom was calculated. The average of standard deviation for relative permittivity and conductivity over the frequency range of 0.5–1 GHz for the selected phantoms (the same as those shown in Figure 8) are shown in Table 3.

## 5. Error Analysis

**Figure 9:**
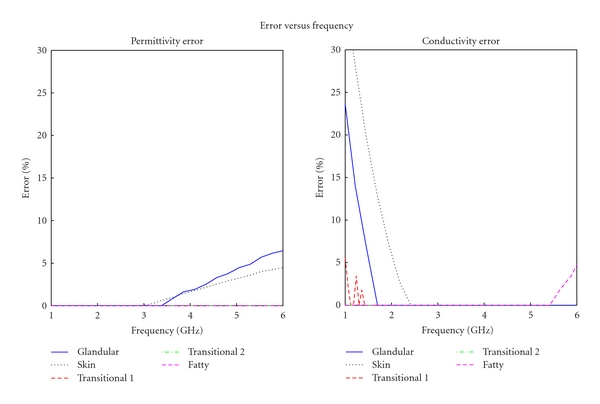
Error for each tissue mimicking phantom.

If the dielectric properties were within the acceptable range of each tissue the error was considered to be zero. The error for each tissue phantom was calculated across the entire frequency spectrum, using (3). The error versus frequency for each tissue phantom is shown in Figure 9. The average error over the frequency band is calculated and the results are shown in Table 4.

(3) $$\begin{array}{cc} {\text{Error}}_{\varepsilon } & =\sqrt{\frac{{\left(\varepsilon_{\text{Debye}}-\varepsilon_{\text{Regression}}\right)}^{2}}{\varepsilon_{\text{Regression}}^{2}}}\\ {\text{Error}}_{\sigma } & =\sqrt{\frac{{\left(\sigma_{\text{Debye}}-\sigma_{\text{Regression}}\right)}^{2}}{\sigma_{\text{Regression}}^{2}}}, \end{array}$$

where

(4) $$\begin{array}{cc} \varepsilon & =\text{Permittivity},\\ \sigma & =\text{Conductivity}. \end{array}$$

Except for a few frequencies, the final phantom mimics the dielectric properties of breast tissues within 10%.

## 6. Procedure

To produce homogeneous mixture each material was measured with amounts prescribed in Table 2. A double boiler was used for heating and a container of ice water was used for cooling the mixtures, when needed. The first step was to mix the measured amount of distilled water with propylene glycol in a container to be placed in the double boiler. Then the temperature of the mixture was raised to 50°C. After the water and propylene glycol reached 50°C the Calf Bloom gelatin was added and mixed in until it is completely dissolved. The result was a clear with a yellow color. While the gelatin was dissolving, the surfactant and formalin were added to the oil and was mixed with the heated solution. Then the solution of water propylene glycol and gelatin was removed from the double boiler. At this point the remaining ingredients were added to the water and propylene glycol.

The mixture has to be continuously stirred until it cools. After it cools outside of the double boiler it should be placed in the ice bath to cool further. Once it was in the ice bath, stirring was continued until the mixture reached 30°C. One should be careful not to stir too vigorously otherwise air bubbles will appear in the mixture and change its dielectric properties. Once the mixture reached 25°C it was poured into containers for molding and, then, refrigerated overnight.

## 7. Heterogeneous Phantom

**Figure 10:**
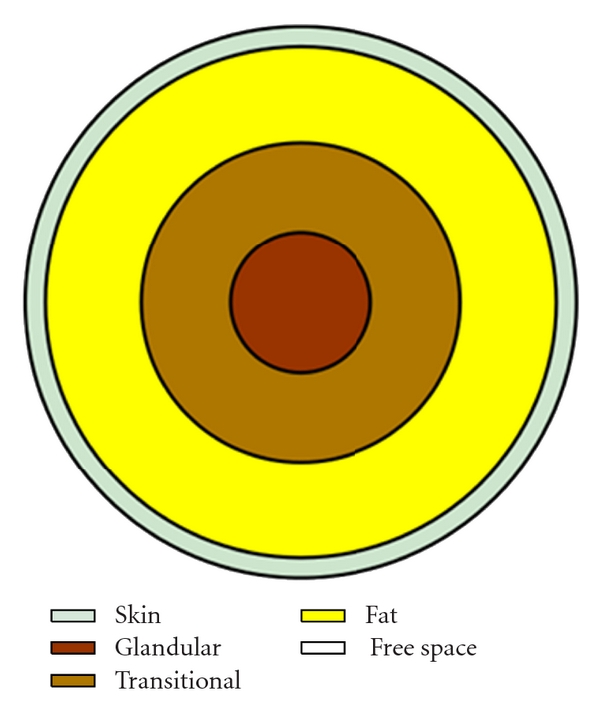
Structure of a heterogeneous phantom.

To accurately assess the microwave imaging techniques, the object of interest must be of a heterogeneous nature. In this paper a simple-layered phantom was created to show the ability to manufacture such a phantom. This phantom is constructed with layers as shown in Figure 10. The mixing process discussed previously was used to produce each tissue type in the phantom and each layer was constructed from the exterior inward to produce a solid object.

**Figure 11:**
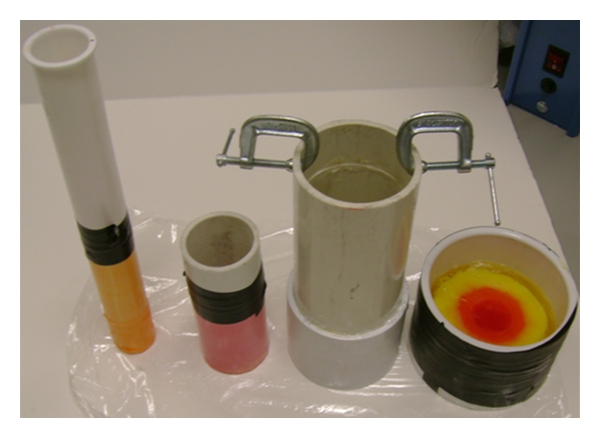
Cylinders used to mold the phantom.

**Figure 12:**
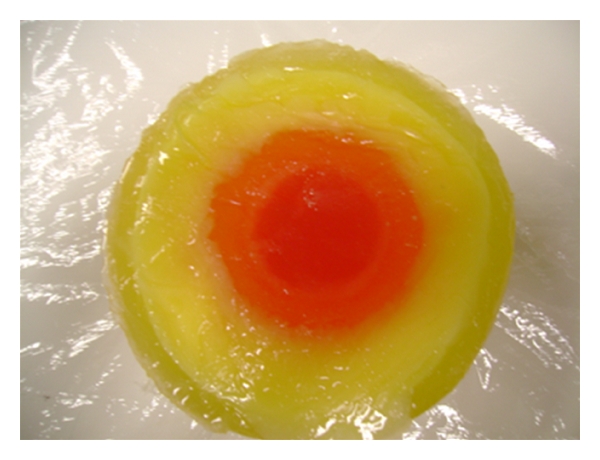
Heterogeneous phantom.

To create a model of the phantom shown in Figure 10, several cylindrical molds were required. To begin a large 6 inch diameter PVC pipe was sealed and used to produce a mold for the exterior. Then a 5 inch diameter PVC pipe was used to form the inner wall for the skin layer. The inner concentric cylinder was removed after the skin phantom was solidified. The mixture for the skin type phantom was then poured and left to cool. After carefully removing the 5 inch diameter PVC pipe, a 3 inch diameter PVC pipe was placed in the middle and fat phantom was poured. The inner cylinder was removed and then a 1.5 inch diameter pipe was placed in the middle of the phantom to make the mold for the transitional phantom. After removing the final pipe the glandular phantom mixture was poured. The molds used for making this phantom are shown in Figure 11 and the final heterogeneous phantom is shown in Figure 12. Food coloring was added to show the different layers in the phantom.

## 8. Phantom Life Study

When using a phantom for benchmarking, it is important to understand how time, humidity, and temperature affect the phantom properties and when the phantom is no longer usefully accurately mimics the tissue. For this reason several measurements of the dielectric properties were taken over the period of a week to better understand the behavior of the proposed phantom mixture over time. The results reported here are only from our initial study. More measurements are required to confirm the repeatability of the results. In addition the changes in dielectric properties when they are formed in a heterogeneous phantom and in contact with different layers should be studied.

**Figure 13:**
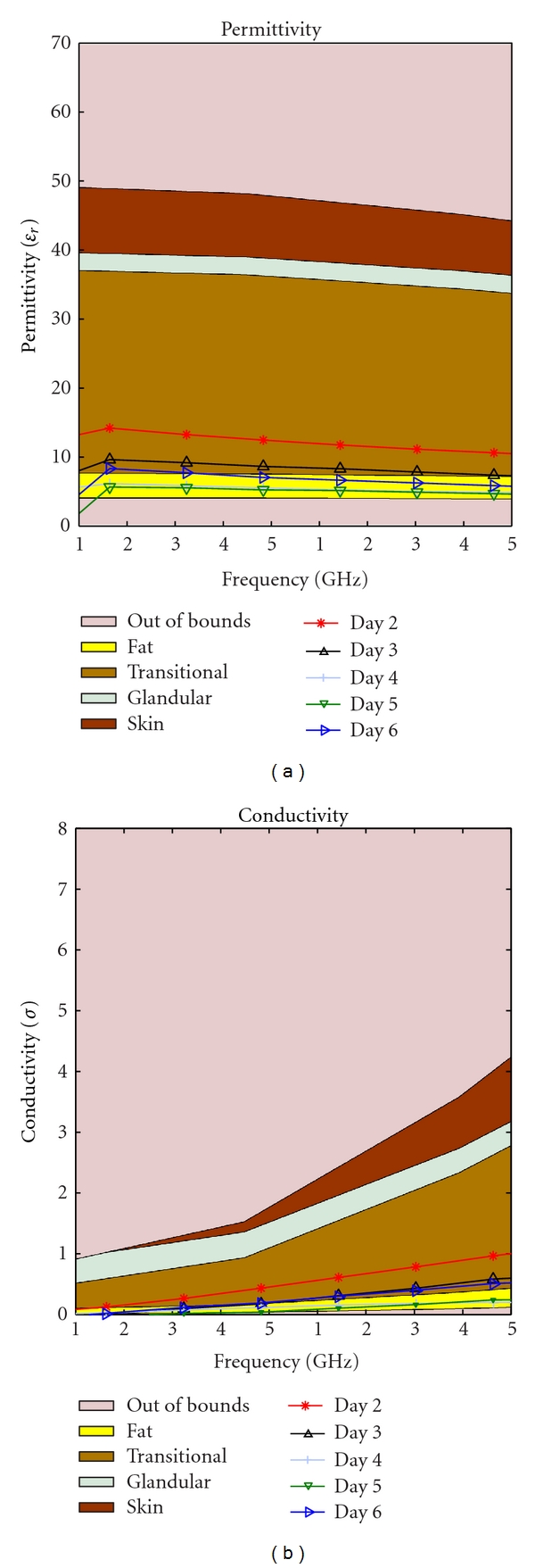
Transitional phantom kept in an open container at a low temperature.

**Figure 14:**
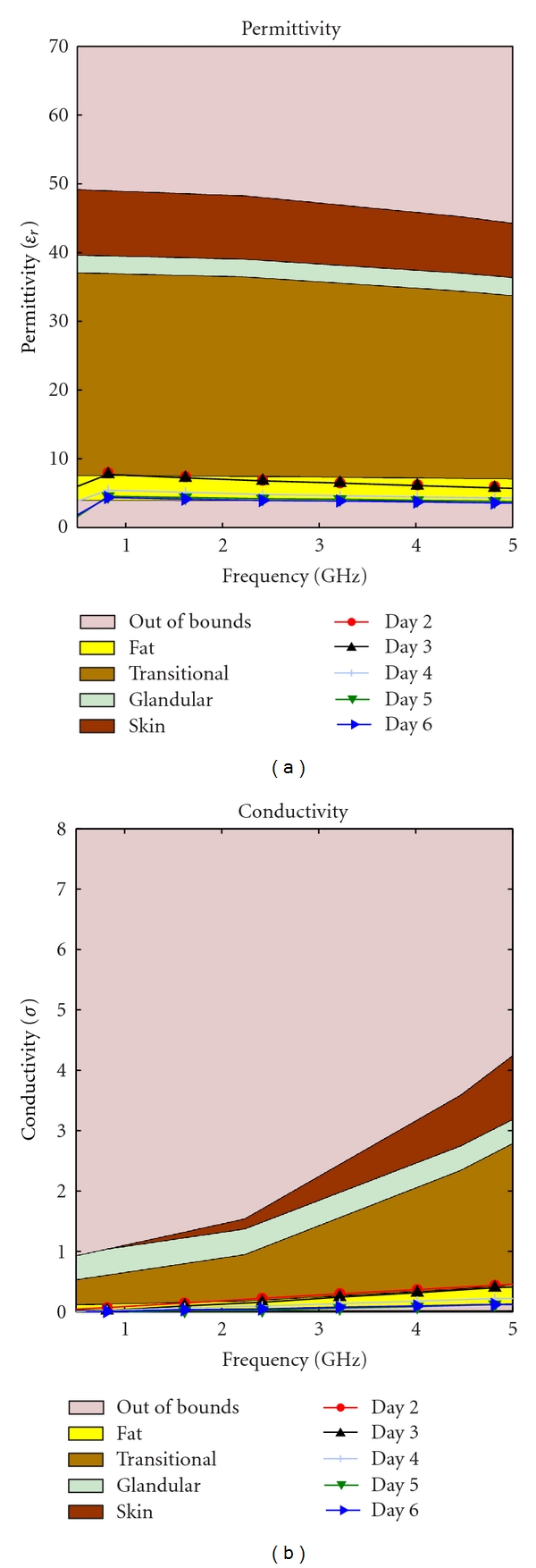
Transitional phantom kept in an open container at room temperature.

**Figure 15:**
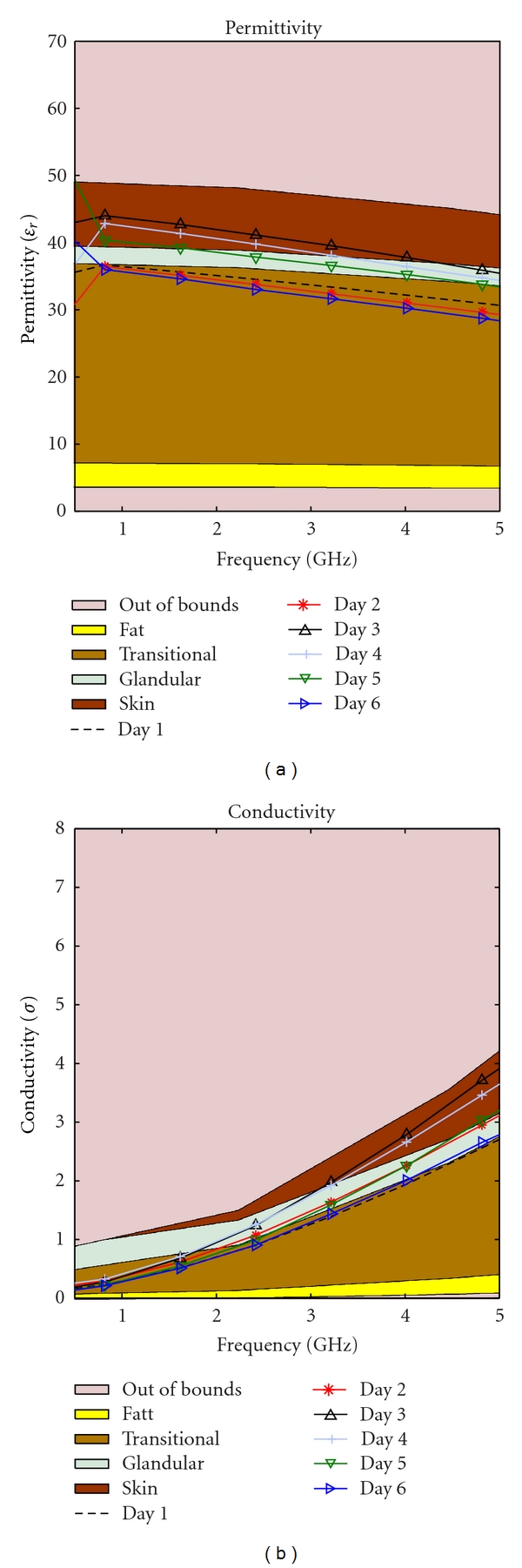
Transitional phantom kept in a sealed container at a low temperature.

**Figure 16:**
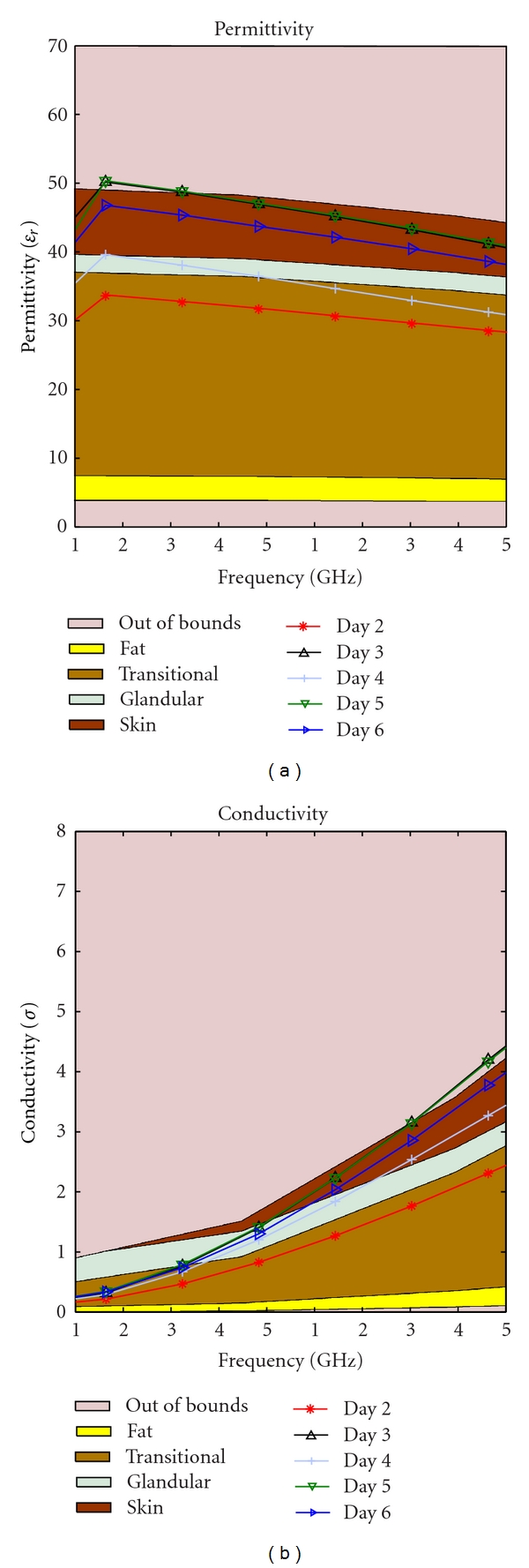
Transitional material kept in a sealed container at a room temperature.

Our hypothesis was that as the phantom ages, the water content decreases due to evaporation. This should cause a drop in the permittivity over time. This change is inevitable, but to slow the evaporation two different storage techniques were tested: storing in low temperature and keeping the phantom in a closed container. Four identical phantoms were needed to start the study. The identical mixtures were obtained by creating one large mixture then dividing these into four different pieces for testing. Different methods of storage produced strikingly different results as depicted in Figures 13–16.

As time passed the permittivity of each sample decreased, as it was expected. Additionally, we found that if the material is placed in a sealed container the dielectric properties can change dramatically, this could be due to a change in partial pressure, removing some gas from the mixture and thereby causing the permittivity to rise. This phenomenon can be observed by comparing Figure 13 with Figure 15, as well as Figure 14 with Figure 16.

In conclusion, the dielectric properties seem more stable if the phantom is kept in a refrigerator, but not in a vacuum container. It should be noted that the phantom properties show substantial changes with respect to temperature, before using a phantom it is important to allow it to return to the room temperature. Under these conditions it appears that this phantom should be useful for up to 5 days.

## 9. Conclusion

We proposed a systematic approach to building tissue phantoms based on regression models. This process was used to form a mixture that adequately mimics different breast tissues with respect to their dielectric properties. This resulting phantom is made of a mixture that can be molded with many traditional molding techniques. By using a combination of molding techniques, one can adequately represent the physical and electrical properties of human breast. The results shown in this paper are based on the available breast tissue dielectric properties from *ex vivo* measurements; therefore, the comparison is performed at room temperature. As a future study the phantom behavior at higher temperature (closer to body temperature) should be compared with *in vivo* tissue properties, once they become available.

## Figures and Tables

**Table 1:** Regression table.

Data sets used in regression analysis				
Test number	Water (g)	Oil (g)	Propylene glycol (g)	Gelatin (g)
1	80	50	7	10
2	40	50	7	10
3	80	10	7	10
4	40	10	7	10
5	80	50	2	10
6	40	50	2	10
7	80	10	2	10
8	40	10	2	10
9	80	50	7	5
10	40	50	7	5
11	80	10	7	5
12	40	10	7	5
13	80	50	2	5
14	40	50	2	5
15	80	10	2	5
16	40	10	2	5

**Table 2:** Mixture recipes.

	Phantom type			
Material	Skin	Fibroglandular	Transitional	Fatty
Distilled water	80.00 g	80.00 g	40.00 g	40.00 g
Safflower oil	14.00 g	21.00 g	13.00 g	39.00 g
Propylene glycol	7.00 g	7.00 g	5.88 g	2.00 g
200 bloom calf-skin gelatin	5.88 g	5.00 g	5.00 g	7.00 g
Formalin (37% formaldehyde solution)	0.30 g	0.30 g	0.30 g	0.30 g
Surfactant	0.30 g	0.30 g	0.30 g	0.30 g

**Table 3:** Average of standard deviation (STD) for selected phantoms shown in Figure 8 over frequency range of 0.5–6 GHz.

Phantom type	Average of STD for *ε*_r_	Average of STD for *σ*
Fat	2.015	0.056
Transitional 1	3.372	0.252
Transitional 2	0.312	0.030
Skin	3.921	0.931
Glandular	7.029	0.478

**Table 4:** The average and standard deviation of error of different phantoms.

	Average error		Standard deviation of error	
	Permittivity	Conductivity	Permittivity	Conductivity
Fatty tissue	4.40%	0.02%	0.0570	0.1126
Skin	7.18%	6.32%	0.1421	0.0389
Fibroglandular tissue	2.99%	11.74%	0.1160	0.0822
Transitional tissue 1	0.16%	0.00%	0.0000	0.0000
Transitional tissue 2	0.00%	0.00%	0.0077	0.0000

## References

[1] Salvador SM, Vecchi G (2009). Experimental tests of microwave breast cancer detection on phantoms. *IEEE Transactions on Antennas and Propagation*.

[2] Sill JM, Fear EC (2005). Tissue sensing adaptive radar for breast cancer detection-experimental investigation of simple tumor models. *IEEE Transactions on Microwave Theory and Techniques*.

[3] Meaney PM, Yagnamurthy NK, Paulsen KD (2002). Pre-scaled two-parameter Gauss-Newton image reconstruction to reduce property recovery imbalance. *Physics in Medicine and Biology*.

[4] Klemm M, Leendertz JA, Gibbins D, Craddock IJ, Preece A, Benjamin R (2009). Microwave radar-based breast cancer detection: imaging in inhomogeneous breast phantoms. *IEEE Antennas and Wireless Propagation Letters*.

[5] Ostadrahimi M, Reopelle R, Noghanian S, Pistorius S, Vahedi A, Safari F A heterogeneous breast phantom for microwave breast imaging.

[6] Mashal A, Gao F, Hagness SC (2011). Heterogeneous anthropomorphic phantoms with realistic dielectric properties for microwave breast imaging experiments. *Microwave and Optical Technology Letters*.

[7] Lazebnik M, Madsen EL, Frank GR, Hagness SC (2005). Tissue-mimicking phantom materials for narrowband and ultrawideband microwave applications. *Physics in Medicine and Biology*.

[8] Schroeder MJ, Anupama S, Nelson RM (2008). An analysis on the role of water content and state on effective permittivity using mixing formulas. *Journal of Biomechanics, Biomdical, and Biophysical Engineering*.

[9] Bao JZ, Swicord ML, Davis CC (1996). Microwave dielectric characterization of binary mixtures of water, methanol, and ethanol. *Journal of Chemical Physics*.

[10] William N (2010). *Principles of Statistics for Engineers and Scientists*.

[11] Montgomery DC, Runger GC, Hubele NF (2007). *Engineering Statistics*.

[12] Lazebnik M, McCartney L, Popovic D (2007). A large-scale study of the ultrawideband microwave dielectric properties of normal breast tissue obtained from reduction surgeries. *Physics in Medicine and Biology*.

[13] Gabriel S, Lau RW, Gabriel C (1996). The dielectric properties of biological tissues: III. Parametric models for the dielectric spectrum of tissues. *Physics in Medicine and Biology*.

[14] Minitab Inc. http://www.minitab.com/en-US/default.aspx.

[15] Mathworks http://www.mathworks.com/products/matlab/.

